# Simplified method using kidney / ureter / bladder x-ray to determine the appropriate length of ureteral stents

**DOI:** 10.1590/S1677-5538.IBJU.2017.0620

**Published:** 2018

**Authors:** Makoto Taguchi, Kenji Yoshida, Motohiko Sugi, Hidefumi Kinoshita, Tadashi Matsuda

**Affiliations:** 1 Department of Urology and Andrology, Kansai Medical University, Osaka, Japan

**Keywords:** Kidney, Ureter, Urinary Bladder

## Abstract

**Purpose:**

To investigate a method to determine the appropriate length of ureteral stents, given that the stent length may lead to exacerbation of urinary symptoms if the stent crosses the bladder midline.

**Materials and Methods:**

We retrospectively reviewed the position of the distal curl of the ureteral stent using kidney/ureter/bladder (KUB) radiographs after ureteroscopic lithotripsy in 165 patients who underwent placement of 24- or 26-cm ureteral stents. According to the KUB findings, we categorized the position of the distal curl of the ureteral stent into two groups. In Group 1, the stents did not cross the midline (appropriate length); in Group 2, the stents crossed the midline (inappropriate length). We assessed several patient parameters (sex, height, body mass index, and stone side) and the index of ureteral length using KUB radiographs (“C-P”) and computed tomography (CT, “P-V”). Multivariate analysis was performed to identify the most significant factors affecting the position of ureteral stents. We also calculated the cutoff points of the receiver operating characteristic (ROC) curve of C-P and P-V for the position of ureteral stents.

**Results:**

The multivariate analysis showed that C-P was the most significant factor affecting the position of ureteral stents (p < 0.001) in patients with 24- and 26-cm ureteral stents. Comparison of the ROC curves of C-P and P-V showed that C-P was superior to P-V (p < 0.01) in patients with 24- and 26-cm stents.

**Conclusion:**

The use of KUB radiographs was effective and simple in determining the appropriate length of ureteral stents.

## INTRODUCTION

Since Zimskind et al. (1) introduced ureteral stents in 1967, such stents have become widely used for the maintenance of renal function, pain relief, and the treatment of urinary tract infections. However, many complications of ureteral stenting have been reported, such as incomplete emptying, bladder pain, frequency, hematuria, and migration. In one study, ureteral stenting reportedly decreased the urination-related quality of life (QOL) in 80% of patients who underwent ureteral stenting (2). Several factors have been investigated for their effects on ureteral stent - related symptoms, including stent length, (3, 4) diameter, (5-7) material, (7) softness, (8) position, (9) and loop completeness (3). Among these factors, determination of the most appropriate ureteral stent length assumes importance in reducing stent-related complications. Some studies have revealed that placement of overly long ureteral stents that cross the bladder midline can lead to worsening of urinary symptoms (3, 10, 11). Therefore, we consider the position of the ureteral stent to be an important factor in stent-related surgery. In the present study, we evaluated a method to determine the appropriate ureteral stent length and ensure that the stent does not cross the bladder midline.

The optimal method for determining the appropriate ureteral stent length remains unclear. In previous reports, the appropriate ureteral stent length for each patient was calculated by three different methods. The first is direct measurement of the ureter itself using a guide wire or ureteral catheter (12-16). The second involves measurement of the distance from the pelviureteric junction (PUJ) to the vesicoureteric junction (VUJ) by either retrograde or intravenous pyelography (16-19). The third method provides an estimation of the appropriate stent length using a formula based on the patient's height. The patient's height is reportedly a more reliable guide for obtaining an appropriate ureteral stent length than direct ureteral measurement using a guide wire and ureteral catheter (12, 13, 15, 16, 18, 19). However, there is no standard and simplified method for determining the appropriate ureteral stent length that prevents a decline in urination-related QOL. Moreover, in some hospitals, assorted lengths of ureteral stents are not stocked, and preoperative prediction of ureteral stent lengths is often needed. In this study, therefore, we measured the distance between two points on a kidney / ureter / bladder (KUB) radiograph using retrospective data and evaluated predictors to place ureteral stents (of lengths 24 and 26 cm) so as not to cross the bladder midline. We have developed a predictive and simplified method for determination of the appropriate length of ureteral stents using KUB radiographs with the aim of reducing urination-related symptoms and concomitant QOL.

## MATERIALS AND METHODS

### Study population

This study was approved by our institutional review board (authorization number: H160741). From January 2013 to December 2015, 168 of 204 patients who underwent ureteroscopic lithotripsy and ureteral stent insertion were enrolled. At the end of the procedure, each patient underwent placement of a ureteral stent (Inlay Optima; C.R. Bard Inc., NJ, USA or Polaris Ultra; Boston Scientific, MA, USA). The diameter of all ureteral stents was 6F and the length was 24 or 26 cm according to the surgeon's discretion. All the stents were placed with full curls in the bladder and kidney.

**Figure 1:**
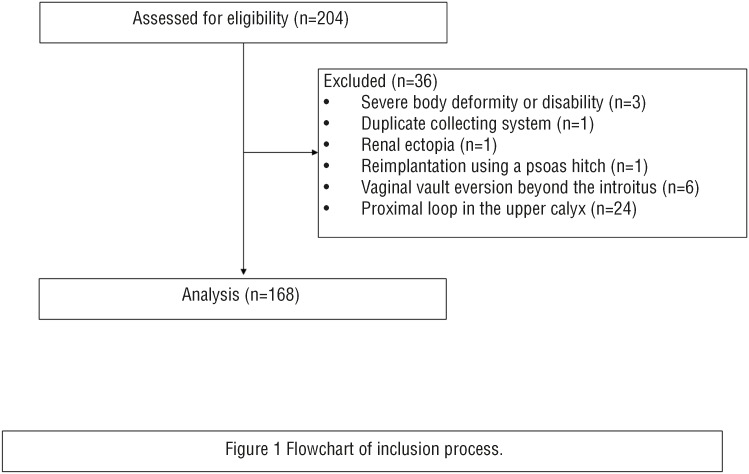
Flowchart of inclusion process.

The exclusion criteria were severe body deformity or disability, a duplicate collecting system, renal ectopia, reimplantation using a psoas hitch, vaginal vault eversion beyond the introitus, and a proximal loop in the upper calyx (Figure-1).

### Patient parameters

We assessed several parameters to evaluate the correlation between these characteristics and the appropriate ureteral stent length. Patient demographics including age, gender, height, body weight, body mass index (BMI), and stone side were reviewed. We calculated the length as the index to choose appropriate ureteral stent length for not crossing the bladder midline using KUB radiographs and computed tomography (CT).

### Measurement of index using KUB radiographs

**Figure 2:**
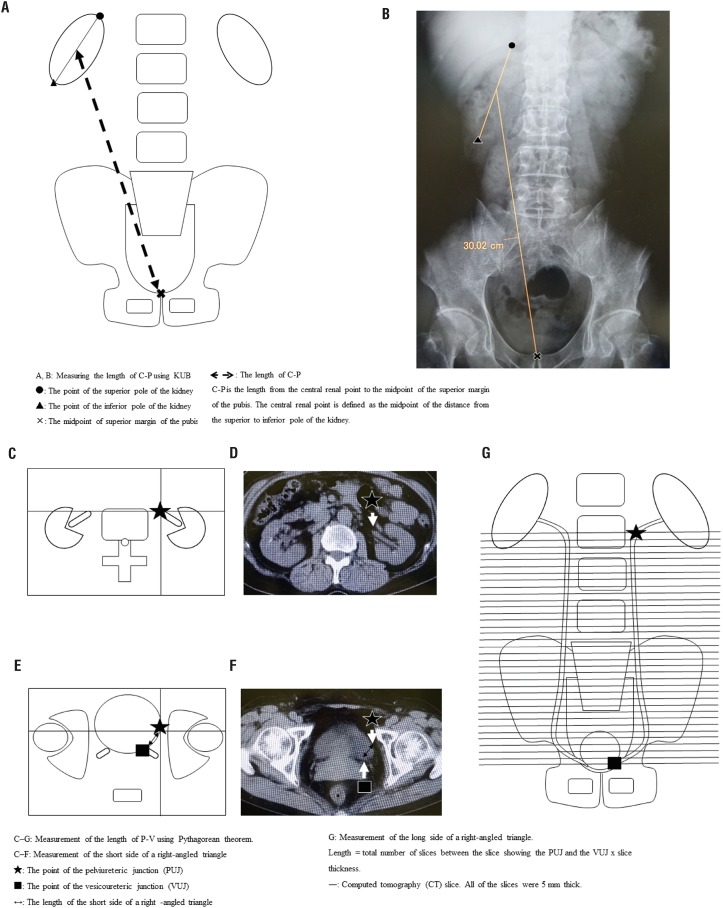
(A-G) - Measuring the length of C-P and P-V. **A, B - C-P** is the length from the central renal point to the midpoint of superior margin of the pubis. Central renal point is defined as the midpoint of distance from extremitas superior renis to extremitas inferior renis. / **C, D, E, F, G** - Measuring the length of P-V using Pythagorean theorem. / **C, D, E, F** - Measuring the short side of a right-angled triangle / **G** - Measuring the long side of a right-angled triangle. / **It length** = the total number of slices between the slice showing the PUJ and the VUJ × slice thickness.

We measured the index using preoperative KUB films. KUB filming conditions were standardized at maximum inspiration in the supine position, and imaging was performed at 70 kV and 132 mA. The index used in this study was the length from the central renal point to the midpoint of the superior margin of the pubis (C-P), measured on KUB films (Figures 2A and B). The central renal point was defined as the midpoint of the distance from the extremitas superior renis to the extremitas inferior renis.

### Measurement of index using CT

All patients were scanned with a 64-slice CT scanner (120 kV, 200 mA, and 5-mm slice thickness). We also calculated the length from the PUJ to the VUJ (P-V) using CT and the Pythagorean theorem and compared this method with the above-described method to determine which more effectively predicts the appropriate ureteral stent length. The CT index was calculated using Carestream Vue PACS (Carestream Health, Rochester, NY, USA), and all CT images were reviewed by a single urologist (M.T.) with 5 years of experience as an urologist. First, in the CT slice showing the PUJ, we marked the point of the PUJ (Figures 2C and D, star). Next, in the CT slice showing the VUJ, we marked the corresponding point for the PUJ slice (Figures 2E and F, star) and measured the distance from the VUJ (Figures 2E and F, square) to the marked point (Figures 2E and F, star) in the CT slice showing the VUJ. We defined this length as the short side of a right-angled triangle (Figures 2E and F; from star to square). We then defined the length of the long side of a right-angled triangle, calculated by the total number of slices between the slice showing the PUJ (Figure-2G, star) and the VUJ (Figure-2G, square). All slices were 5 mm thick (Figure-2G). Finally, we calculated the length of P-V using the Pythagorean theorem ([P - V]2 = [short side] 2 + [long side] 2).

### Definition of appropriate ureteral stent length

**Figure 3:**
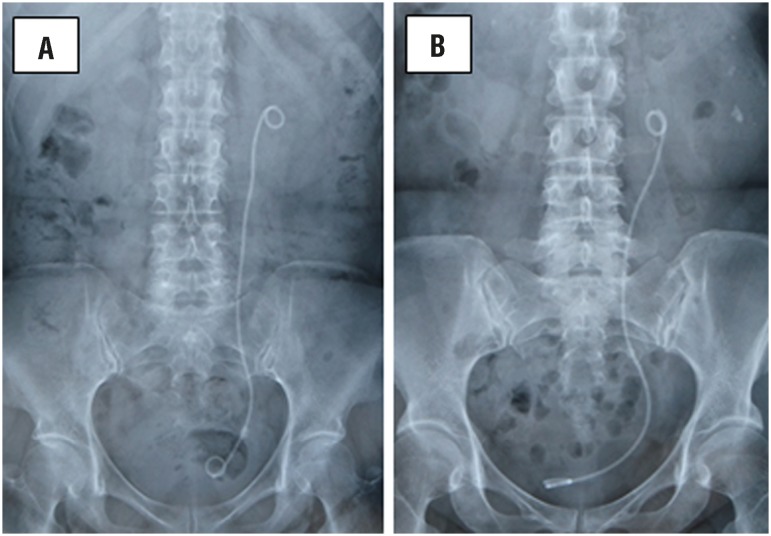
(A, B) - Classification of the intravesical ureteral stent position. (A) Not crossing midline (Group 1). (B) Crossing midline (Group 2).

We routinely obtained KUB films to confirm the presence of residual stones on postoperative day 1. We retrospectively reviewed the position of the ureteral stents using these KUB films. All KUB films were reviewed by a single urologist (M.T.). We categorized the patients into two groups according to the position of the distal curl of the ureteral stent on the KUB films using the technique described by Giannarini et al. (11) In Group 1, the stent did not cross the midline (appropriate length of ureteral stent, Figure-3A); in Group 2, the stent crossed the midline (inappropriate length of ureteral stent, Figure-3B).

### Statistical analysis

In each group, both of which included patients with 24- and 26-cm ureteral stents, we evaluated the correlation between the position of the ureteral stents and various patient parameters: age, gender, height, body weight, BMI, stone side, C-P, and P-V. Univariate analysis was performed using either the Mann-Whitney U-test or the χ2 test to evaluate the correlation between the position of the ureteral stents and patient parameters. Multivariate analysis was performed using a logistic regression model to identify the most significant factors affecting the position of the ureteral stents. Furthermore, we calculated the cutoff points of the receiver operating characteristic (ROC) curve, area under the ROC curve (AUROC), and 95% confidence interval (CI) of the C-P and P-V for the position of the ureteral stents. Data were analyzed using the IBM SPSS Statistics V21.0 software package.

## RESULTS

Table-1 shows the patients’ demographic data. In Group 1, 46 (59.0%) and 46 (51.1%) patients had 24- and 26-cm indwelling stents, respectively. In Group 2, 32 (41.0%) and 44 (48.9%) patients had 24- and 26-cm indwelling stents, respectively.

**Table 1:** The demographic data of patients with indwelling 24-cm and 26-cm ureteral stents.

		24 cm ureteral stents	26 cm ureteral stents
		n (%) or median (range)	
Patients		78	90
Age (years)		62.5 (92-33)	55 (26-84)
**Gender**			
	male	29 (37.2)	85 (94.4)
	female	49 (62.8)	5 (5.6)
Height (m)		1.57 (1.39-1.81)	1.65 (1.45-1.85)
Body weight (kg)		59.2 (30.2-118.2)	63.3 (39-108.6)
BMI (kg/m^2^)		24.2 (15.4-35.2)	23.7 (17.8-36.1)
**Stone side**			
	left	52 (66.7)	58 (64.4)
	right	26 (33.3)	32 (35.6)
**Ureteral stent position**			
	crossing midline	32 (41.0)	44 (48.9)
	not crossing midline	46 (59.0)	46 (51.1)

**BMI** = body mass index.

Table-2 shows the results of the univariate and multivariate analyses performed to evaluate the correlation between the position of the ureteral stents and patient parameters. Comparison of Groups 1 and 2 using univariate analysis revealed no significant differences in age, gender, height, body weight, BMI, or stone side in either the 24- or 26-cm group. However, C-P and P-V were significantly longer in Group 1 (not crossing midline) than Group 2 (crossing midline) in both the 24- and 26-cm groups (p < 0.001).

**Table 2A:** Multivariate analysis of patients with 24 cm ureteral stents.

		Group 1 (Not crossing midline)	Group 2 (Crossing midline)	Univariate analysis^a^	Multivariate analysis^b^		
				p-value	p-value	OR	95% CI
**Gender**							
	male	17 (37.0)	12 (37.5)	0.98			
	female	29 (63.0)	20 (62.5)				
Height (m)		1.59 (1.41-1.81)	1.57 (1.39-1.78)	0.69			
BMI (kg/m^2^)		24.3 (17.1-32.0)	23.8 (15.4-35.2)	0.99			
**Stone side**							
	left	31 (67.4)	21 (65.6)	0.92			
	right	15 (32.6)	11 (34.4)				
C-P		28.5 (25.5-33.9)	26.1 (19.9-28.8)	<0.001	<0.001	7.445	2.689-20.612
P-V		20.2 (16.7-25.3)	19.1 (14.7-21.2)	<0.001	0.331	0.966	0.901-1.036

a Mann-Whitney U-test;

b Logistic regression analysis;

**OR =** odds ratio; **CI =** confidence interval

According to our multivariate analysis, C-P was the most significant factor affecting the position of the ureteral stents in both the 24- and 26-cm groups (p < 0.001 for both) (Tables 2A and B, respectively).

**Table 2B:** Multivariate analysis of patients with 26 cm ureteral stents.

	Group 1 (Not crossing midline)	Group 2 (Crossing midline)	Univariate analysis^a^	Multivariate analysis^b^		
			p-value	p-value	OR	95% CI
Gender						
male	42 (91.3)	43 (97.7)	0.18			
female	4 (8.7)	1 (2.3)				
Height (m)	1.66 (1.47-1.80)	1.64 (1.45-1.85)	0.24			
BMI (kg/m^2^)	23.5 (17.8-36.1)	24.1 (19.7-35.2)	0.40			
Stone side						
left	29 (63.0)	29 (65.9)	0.78			
right	17 (37.0)	15 (34.1)				
C-P	30 (26.0-34.8)	28.2 (24.6-29.9)	<0.001	<0.001	3.003	1.701-5.301
P-V	21.0 (17.7-27.1)	19.5 (17.4-22.3)	<0.001	0.273	1.018	0.986-1.051

a Mann-Whitney U-test;

b Logistic regression analysis;

**OR** = odds ratio; **CI** = confidence interval

**Figure 4:**
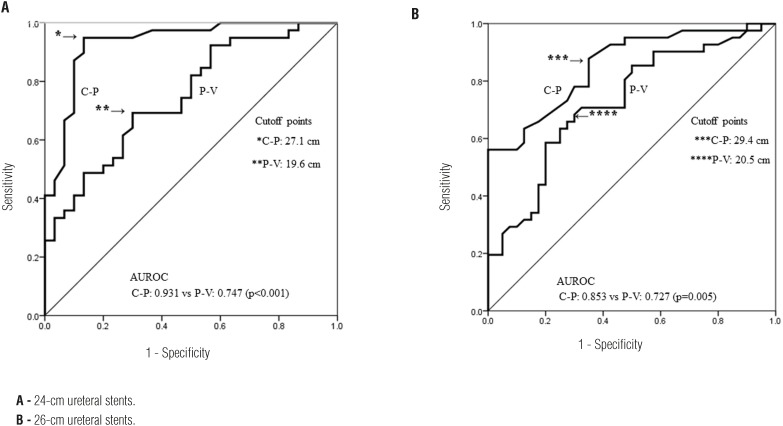
(A, B) - Receiver operating characteristic curves for success of ureteral stenting of KUB and CT, and area under the receiver operating characteristic curve (AUROC).

Figure-4 shows the comparison of the ROC curves of C-P and P-V and the AUROC in the patients with 24- and 26-cm ureteral stents. The cutoff points of the ROC curve of C-P and P-V in the patients with 24-cm ureteral stents were 27.1 and 19.6 cm, respectively, and those in the patients with 26-cm stents were 29.4 and 20.5 cm, respectively. Comparison of the ROC curves of C-P and P-V showed that C-P was superior to P-V in both the 24- and 26-cm groups (p < 0.01).

## DISCUSSION

Determination of the appropriate ureteral stent length is very important for reducing stent-related complications. However, few reports have addressed this topic, and no guidelines regarding ureteral stents exist. In this study, we evaluated several patient parameters that we considered relevant when choosing the appropriate stent length. We found that the method using KUB films was more useful and simpler than the methods using patient height or CT for determining the appropriate stent length. We measured the C-P length using KUB films. In this study, we found that a 26-cm ureteral stent is appropriate for patients with a C-P of ≥ 29.4 cm, that a 24-cm ureteral stent is appropriate for patients with a C-P of 27.1 to < 29.4 cm, and that a 22-cm ureteral stent may be appropriate for patients with a C-P of < 27.1 cm. The appropriate ureteral stent length was short if the C-P length was shorter. Because we considered that a short C-P or P-V means that the ureter length is also expected to be short, the appropriate ureteral stent length was also short.

Some studies have reported that crossing of a ureteral stent over the bladder midline may lead to worsening of urinary symptoms (3, 10, 11). Rane et al. (3) investigated the correlation between the position of the ureteral stent and stent-related symptoms in 60 patients and reported that a ureteral stent that crosses the bladder midline causes significantly more frequency and urgency. Ho et al. (10) evaluated whether the ureteral stent length affects stent-related symptoms after placement of stents in 87 patients. They discovered that the ureteral stent length was associated with the position of the distal loop of the stent and reported that a longer stent crossing the bladder midline causes more irritative symptoms. Giannarini et al. (11) assessed the predictors of morbidity in 84 patients with indwelling ureteral stents. Using multivariate analyses, they reported that the location of the distal loop of the ureteral stent (not crossing the bladder midline) had the strongest association with ureteral stent-related symptoms. Therefore, we consider that crossing of a ureteral stent over the bladder midline may lead to worsening of urinary symptoms and that choosing the most appropriate ureteral stent length for each patient is important to improve stent-related symptoms.

Table-3 shows reported clinical studies to choose the appropriate ureteral stent length not crossing the bladder midline. Pilcher and Patel (13) reported that the patient's height is a more reliable guide to choosing the most appropriate ureteral stent length than is direct ureteral measurement using a guide wire and ureteral catheter. They compared the accuracy of a patient height-based formula for choosing the correct ureteral stent length with that of direct ureteral length measurement. In their study, the patient's height correctly predicted the appropriate stent length in the majority of ureters, and direct ureteral measurement oversized the ureteral stent length in 83% of cases (13). Additionally, Ho et al. (20) found a 22-cm ureteral stent to be more appropriate for patients of < 175 cm in height, who comprised nearly 90% of their study population. Lee et al. (21) also reported that a 22-cm stent was appropriate for patients of < 175 cm in height. Conversely, Jeon et al. (16) found direct measurement of the ureteral length to be a more reliable method than determination of the stent length according to patient height. Wills et al. (17) reported that measurement of the ureteral length by intravenous urography is useful. However, this method requires a full-length intravenous urography film, and tracing the curved ureter viewed on a retrograde or intravenous pyelography film is difficult (21). Therefore, we considered that establishment of a simple method with which to determine the appropriate ureteral stent length was necessary and recommend the herein-described method using KUB films, which we consider more useful and simpler than other methods.

**Table 3:** Clinical studies performed to choose the appropriate ureteral stent length that does not cross the bladder midline.

Study	n	Methods to choose stents	Outcome
Pilcher and Patel (13)	41	Ureteral catheter vs patient's height	Patient's height was a more reliable guide.
Ho et al. (20)	408	Comparing patient's height and stent position	Patient's height could predict the ideal stent length.
Lee et al. (21)	70	Comparing patient's height and stent position	A 22 cm ureteral stent was appropriate for Korean patients smaller than 175 cm in height.
Jeon et al. (16)	70	Direct measurement using guidewire vs patient's height	Direct measurement of ureteral length using guidewire was easy and reliable. Patient's height did not correlate well with appropriate ureteral length.
Wills et al. (17)	40	Comparing with the ideal stent length and the length of the ureter measured on intravenous urography	Measuring on intravenous urography had the correlation with the ideal stent length.
Barrett et al. (22)	59	Patient's height vs L1-L5 height vs length measured on CT	CT measurements could be used to choose the appropriate stent length.
Our study	168	Comparing predictors (sex, patient's height, BMI, side, KUB radiograph, CT) to determinate the appropriate length of ureteral stent.	KUB radiograph and CT were significant factor affecting the position of the ureteral stents according to our multivariate analysis.

**CT** = Computed tomography, **KUB** = kidney/ureter/bladder

Barrett et al. (22) reported using CT to choose the most appropriate stent length; in this technique, the ureteral length can be measured by identifying the location of the ureter in each CT slice. We referred to this method to measure the index using CT in this study. However, this method requires considerable time and effort. Moreover, CT has some limitations such as radiation exposure, measurement error associated with slice thickness, and the need for precise measurement using rendering software. Furthermore, the location of the ureteral orifices differs according to whether bladder filling is performed, (23) although bladder filling was not a standard of care in the present study. Therefore, we consider that we should investigate a more useful method than CT to choose the appropriate ureteral stent length.

This report is the first to calculate cutoff points for determination of the appropriate length of ureteral stents. We have herein introduced our method using KUB films, which is inexpensive and less invasive.

This study has some limitations. First, it was a retrospective and non-randomized trial, and the choice of the ureteral stent was entirely dependent upon the operator. Second, we did not standardize the type of ureteral stents, and the coiling patterns varied among the stents. Third, we did not use 22- and 28-cm ureteral stents and thus did not evaluate the appropriate C-P length for stents of these lengths. Fourth, we did not evaluate the patient's ureteral stent-related symptoms. Future studies should involve reassessment using a 22-cm ureteral stent and evaluation of ureteral stent-related symptoms. Fifth, the method of measurement of the index using CT did not use the coronal plane, and the method using the Pythagorean theorem might be complicated. If we use other methods when measuring the index using CT, there would be a possibility that CT is superior to KUB. Therefore, it is controversial whether these parameters could be transposed to tomography. Finally, the renal shadow was occasionally unclear because of bowel gas. Therefore, some preoperative KUB films were seldom needed. In this study, we could measure the index of all patients using KUB films because we obtained some KUB films as a preoperative assessment, and only one or two films were needed to measure the index in most cases. Furthermore, all KUB films were reviewed by a single urologist and we have not confirmed whether other urologists can measure the index using KUB. We do not consider these methods to be complicated. However, future studies should involve reassessment in multiple centers.

## CONCLUSIONS

We consider that our method using KUB radiographs is useful and simple to determine the appropriate ureteral stent length. Furthermore, we can preoperatively choose an appropriate ureteral stent length compared with direct ureteral measurement using a guide wire and ureteral catheter. However, this study has some limitations and we could not conclude that the method of measurement of the index using KUB is superior to CT.

## ABBREVIATIONS

KUB: = kidney, ureter, bladder X-ray
ROC: = receiver operating characteristic
QOL: = quality of life
PUJ: = pelviureteric junction
VUJ: = vesicoureteric junction
BMI: = body mass index
CT: = computed tomography
AUROC: = area under the receiver operating characteristic curve
CI: = confidence interval
